# INTEREST GROUP SESSION—INTERNATIONAL AGING AND MIGRATION: AGING SOCIETY: INDIVIDUAL, FAMILY, AND COMMUNITY COLLABORATION FROM A GLOBAL PERSPECTIVE

**DOI:** 10.1093/geroni/igz038.1373

**Published:** 2019-11-08

**Authors:** Noriko Toyokawa, Vivian W Lou

**Affiliations:** 1 California State University San Marcos, San Marcos, California, United States; 2 The University of Hong Kong, Hong Kong, P.R.C., Hong Kong

## Abstract

The purpose of this symposium is two-fold: (1) to promote mutual understanding and communication regarding the long-term caregiving plans for older immigrants/refugees between policymakers and researchers in the fields of behavioral and social sciences and (2) to discuss the needs for connecting local needs of older immigrants/refugees and their families with a global plan for aging society. The symposium is structured by three empirical studies on older adults and their caregivers by behavioral and social sciences researchers, followed by a presentation of the needs for an international convention on the rights of older people by an advocate in the global network to promote older people’s health. Miyawaki and colleagues focus on the residential status quo, family relations, and prevalence of chronic diseases among older Vietnamese refugees in Houston, TX, U.S.A. Liu’s qualitative study on Taiwanese professional caregivers’ perceptions of clients in adult daycare services reveals the relation between staff’s negative image of aging and their practice. Toyokawa conceptualizes middle-aged Mexican immigrants’ sense of family obligation, as their obligation for reducing children’s caregiving burden and the endorsement predicts their well-being. Three presenters point out the need for standards for basic needs of refugees/immigrants, staff training, and the quality of long-term care, and discuss the meaning of culturally sensitive support based on their studies. Finally, Marumoto advocates the need for an international convention of the rights of older people and standardization of the quality of long-term care. Specific approaches to ‘harness the network’ between local and global efforts are discussed.

